# Simultaneous magnetic resonance diffusion and pseudo‐diffusion tensor imaging

**DOI:** 10.1002/mrm.26840

**Published:** 2017-07-16

**Authors:** Meghdoot Mozumder, Leandro Beltrachini, Quinten Collier, Jose M. Pozo, Alejandro F. Frangi

**Affiliations:** ^1^ Center for Computational Imaging & Simulation Technologies in Biomedicine (CISTIB) Department of Electronic and Electrical Engineering, The University of Sheffield Sheffield UK; ^2^ iMinds Vision Lab Department of Physics, University of Antwerp (CDE) Antwerpen Belgium

**Keywords:** Magnetic resonance imaging, diffusion, perfusion, intravoxel incoherent motion

## Abstract

**Purpose:**

An emerging topic in diffusion magnetic resonance is imaging blood microcirculation alongside water diffusion using the intravoxel incoherent motion (IVIM) model. Recently, a combined IVIM diffusion tensor imaging (IVIM‐DTI) model was proposed, which accounts for both anisotropic pseudo‐diffusion due to blood microcirculation and anisotropic diffusion due to tissue microstructures. In this article, we propose a robust IVIM‐DTI approach for simultaneous diffusion and pseudo‐diffusion tensor imaging.

**Methods:**

Conventional IVIM estimation methods can be broadly divided into two‐step (diffusion and pseudo‐diffusion estimated separately) and one‐step (diffusion and pseudo‐diffusion estimated simultaneously) methods. Here, both methods were applied on the IVIM‐DTI model. An improved one‐step method based on damped Gauss–Newton algorithm and a Gaussian prior for the model parameters was also introduced. The sensitivities of these methods to different parameter initializations were tested with realistic in silico simulations and experimental in vivo data.

**Results:**

The one‐step damped Gauss–Newton method with a Gaussian prior was less sensitive to noise and the choice of initial parameters and delivered more accurate estimates of IVIM‐DTI parameters compared to the other methods.

**Conclusion:**

One‐step estimation using damped Gauss–Newton and a Gaussian prior is a robust method for simultaneous diffusion and pseudo‐diffusion tensor imaging using IVIM‐DTI model. Magn Reson Med 79:2367–2378, 2018. © 2017 The Authors Magnetic Resonance in Medicine published by Wiley Periodicals, Inc. on behalf of International Society for Magnetic Resonance in Medicine. This is an open access article under the terms of the Creative Commons Attribution License, which permits use, distribution and reproduction in any medium, provided the original work is properly cited.

## INTRODUCTION

Diffusion magnetic resonance imaging (dMRI) is a technique that allows mapping of water molecules' movement due to diffusion in biological tissues, in vivo and noninvasively. Water diffusion in biological tissue is constrained by its microarchitecture 1. Hence, with proper modeling techniques, dMRI is capable of capturing several microstructural features and information related to the tissue constituents. There exists several modeling techniques in the literature capable of capturing such information 2, 3, 4, of which the diffusion tensor imaging (DTI) is the most commonly used. In DTI, water diffusion within a voxel is represented with a rank‐2 tensor 5. Although simplistic, this model was shown to be extremely useful for providing meaningful bio‐markers such as mean diffusivity (MD) and fractional anisotropy (FA), that are widely used as measures of microstructural tissue changes 6. DTI is also useful for the analysis of neuronal fiber pathways and their visualization (tractography) 7.

Perfusion MRI, typically performed separately 8, consists of characterizing blood flow in tissues using techniques such as bolus tracking 9 and arterial spin labeling 10. The characterization of blood flow helps in detecting changes in capillary microarchitecture, blood microcirculation, and blood‐tissue exchanges, which are useful for early detection of several disorders 11 including vascular cognitive impairment 12.

The intravoxel incoherent motion (IVIM) technique, originally proposed by Le Bihan et al. 13 captures both diffusion and perfusion phenomena using dMRI. To do so, it utilizes a bi‐exponential model to describe signal attenuation in dMRI with a relatively fast pseudo‐diffusion component (due to blood diffusion and flow) and a relatively slow tissue diffusion component as

(1) $$S=S_{0}\;(f\;\exp(-b\;D^{*})+(1-f)\;\exp(-b\;D)),$$

where **S** is the vector of echo magnitudes of the diffusion weighted signals within a voxel due to the vector of *b*‐values, **b**. The *b*‐value summarizes the attenuating effect of the gradient magnetic field and the diffusion sequence. Here *S*_0_ is the echo magnitude of the diffusion nonweighted signal within a voxel. The diffusion coefficient *D* and pseudo‐diffusion coefficient D* in Equation (1) are scalar quantities. Information related to perfusion can be obtained using the vascular volume fraction (also known as the perfusion fraction), *f*, and pseudo‐diffusion coefficient, *D* ^*^ 14. The IVIM model has been used mainly in abdominal imaging including the liver 15, 16, 17, kidney 18, and the pancreas 19. IVIM studies of the prostate 20, breast 21, and heart 22, are also available. Very recently, IVIM imaging of the human brain has gained importance. Hu et al. reported IVIM parameter changes in brain tumors 23. Federau et al. reported IVIM parameter changes in the brain in reaction to hypercapnia and hyperoxygenation 24, in response to cardiac cycle 25, stroke 26, and in brain tumors 27.

For estimating the IVIM parameters, the dMRI signal can be averaged over a region of interest (19). Region of interest averaging increases the signal‐to‐noise ratio (SNR), effectively yielding more reliable IVIM parameter estimates. However, voxel‐wise information reflecting tissue heterogeneity is lost in this procedure. Also, a skilled radiologist or a medical doctor is required to choose a suitable region of interest by visual inspection. There are however several methods that generate 2D/3D voxel‐wise maps of IVIM parameters 28. These can be grouped under two main categories:

1. **Two‐step method (TSM)**: In this approach, a first step assumes that the dMRI signal at high *b*‐values ( $b\gg 1/D^{*}$) is dominated by diffusion. Using this assumption, Equation (1) reduces to
  (2) $$S_{b‐\text{high}}\approx S_{0}\;(1-f)\;\exp(-b\;D),$$
  where $S_{b‐\text{high}}$ is the dMRI signal at high *b*‐values. The diffusion coefficient *D* and amplitude *S*_0_(1–*f*) are then estimated from Equation (2). In a second step, *D* ^*^ is estimated (typically constraining *D*) using all *b*‐values and the IVIM model, Equation (1). The vascular volume fraction *f* is either estimated in the first step using the fitted *S*_0_(1–*f*) value and experimentally obtained *S*_0_ value 29, or it is estimated in the second step alongside *D* ^*^ 15. TSM estimates are typically computed using nonlinear least squares (NLLS) methods 15, 24, 27. It has been reported that TSM is sensitive to the choice of cut‐off *b*‐value, which in turn depends on the unknown *D* ^*^ values 30.
2. **One‐step method (OSM)**: In this approach all IVIM parameters, (*f*, *D*, *D* ^*^) are simultaneously estimated using Equation (1) 16, 17, 28, 31. OSM estimates are computed either using NLLS methods 16, 28, Markov chain Monte Carlo methods 17, or using wild bootstrap and fusion moves 31. OSM estimation of IVIM parameters is ill‐conditioned 17, and hence suffers from poor reproducibility of the results 16. It has been demonstrated earlier that using priors for the model parameters yields more stable and accurate IVIM parameter estimates 31, 32.

A comparison of TSM and OSM approaches in estimating IVIM parameters is presented in 28, 33. Although the IVIM model 1 is capable of capturing differences in signal attenuation due to blood microcirculation and microstructural water diffusion, it fails to capture the anisotropy of blood vessels and tissue microstructures, whose estimation can be valuable in early detection of several brain disorders, such as dementia 34, schizophrenia, and bipolar disorder 35, which progress with vascular and structural remodeling of the tissues. This anisotropy can be measured as differences in signal attenuation due to direction dependent magnetic field gradients 22.

The study of anisotropy effects observed with the IVIM model was first reported by Callot et al. 36. In this work, direction dependent IVIM parameters (*f*, *D* ^*^, *D*) were estimated with the TSM. Karampinos et al. 37 proposed a modification to IVIM, based on a statistical model of the capillary arrangements and assuming partially coherent laminar flow. Although this model incorporates anisotropy effects due to pseudo‐diffusion, anisotropic diffusion parameters related to tissue microstructures were not estimated. Moreover, it has been recently reported that estimation of such higher‐order metrics could be more vulnerable to experimental design and noise compared to standard tensor metrics (such as FA and MD) 38. Recently a combined intravoxel incoherent motion diffusion tensor imaging (IVIM‐DTI) methodology was proposed, where a combination of the bi‐exponential behavior of IVIM and DTI‐like tensor representation of the vascular signal was used 22. In this model, both pseudo‐diffusion and diffusion were modeled as tensor quantities as opposed to isotropic scalar coefficients as in the IVIM model, Equation (1). Since pseudo‐diffusion is related to perfusion 14, estimating the pseudo‐diffusion and diffusion tensors allow simultaneous imaging of perfusion and DTI. Using IVIM‐DTI, various physiologically relevant parameters obtained separately using DTI and IVIM techniques can be obtained simultaneously. In addition, visualization of pseudo‐diffusion will help in vivo mapping of the vascular orientations and architectures with dMRI, which has not been possible before. A recent IVIM‐DTI study based on TSM estimation of kidney medulla and cortex regions was presented in 39. Given that TSM and OSM methods are either sensitive to cut‐off *b*‐values 30 or initial values 16, there is the need to explore better estimation methods for IVIM‐DTI.

In this article, we perform simultaneous pseudo‐diffusion and DTI using TSM and OSM methods by extending them to IVIM‐DTI parameter estimation. We test the sensitivity of these methods using in silico simulations and in vivo brain imaging data. The TSM and OSM were carried out using the Lavenberg‐Marquardt (LM) algorithm as implemented in MATLAB (R2016a, Mathworks, Natick, MA) since it was the most commonly used method in previous IVIM studies 16, 25, 27, 28. We also tested OSM with a modified NLLS method based on damped Gauss–Newton (DGN), and with the inclusion of a prior for the model parameters 40. DGN method had earlier demonstrated better convergence compared to LM and robustness with respect to initial starting parameters for a nonlinear *ill*‐posed imaging problem in 41.

## METHODS

### Model Description

In this section, we describe the methodology used for generating the dMRI data and fitting of the IVIM‐DTI model, described by 22, 28

(3) $$S=S_{0}\;(f\;\exp(-b\;{\hat{g}}^{T}D^{*}\;\hat{g})+(1-f)\;\exp(-b\;{\hat{g}}^{T}D\;\hat{g})),$$

where $\hat{g}$ is the unit direction vector along which the magnetic field gradient is applied, **D** is the diffusion tensor and D^*^ is the pseudo‐diffusion tensor. The diffusion‐weighted dMRI data, **Y** is thus modeled as

(4) Y=S(X)+e,

where $X=(S_{0},f,D^{*},D)$ is the vector of unknown IVIM‐DTI parameters and *e* is the measurement noise. To enforce positive definiteness on D^*^ and **D**, we parameterized them as 42,

(5) $$\begin{array}{ccc} D^{*}\equiv D^{*}(U)=U^{T}U, & \text{with}\; & U=\left[\begin{array}{ccc} U_{1} & U_{4} & U_{6}\\ 0 & U_{2} & U_{5}\\ 0 & 0 & U_{3} \end{array}\right],\\ D\equiv D(V)=V^{T}V, & \text{with}\; & V=\left[\begin{array}{ccc} V_{1} & V_{4} & V_{6}\\ 0 & V_{2} & V_{5}\\ 0 & 0 & V_{3} \end{array}\right], \end{array}$$

where $U_{i},i=1,\ldots ,6$, and $V_{i},i=1,\ldots ,6$, are the Cholesky components of D^*^ and **D**, respectively. It has been earlier shown that adopting such representation for DTI leads to slightly higher accuracy for estimating MD and FA of **D** in low signal to noise ratio (SNR < 5) and high anisotropic (FA > 0.9) regions 43. Using this representation, the unknown **X** is given by

$$X={\left(S_{0},f,U_{1},\ldots ,U_{6},V_{1},\ldots ,V_{6}\right)}^{T}.$$

### Estimation

We considered four estimation approaches. The first two approaches were extensions of the conventional TSM and OSM to IVIM‐DTI. The third approach is a proposed OSM based on DGN method. The fourth approach is also a proposed OSM DGN method incorporating a prior for the model parameters.

1. TSM: LM estimation of $X_{1}={\left(S_{0}\;(1-f),V_{1},\ldots ,V_{6}\right)}^{T}$ using
  (6) $${\hat{X}}_{1}=\arg\underset{{}_{X_{1}}}{\min}||Y_{b‐\text{high}}-S_{b‐\text{high}}(X_{1})|{|}^{2},$$
  where $Y_{b‐\text{high}}$ is the dMRI signal with $b\geq b_{\text{cut}‐\text{off}}$ and $S_{b-\text{high}}(X_{1})=S_{0}\;(1-f)\;\exp(-b\;{\hat{g}}^{T}\;D\;\hat{g})$ (see Eq. (2)). Then, *f* is calculated using estimated $S_{0}(1-f)$ value and measured *S*_0_ value. Next, estimated $\hat{x}={\left(f,V_{1},\ldots ,V_{6}\right)}^{T}$ is constrained and $X_{2}={\left(U_{1},\ldots ,U_{6}\right)}^{T}$ is estimated using LM as
  (7) $${\hat{X}}_{2}=\arg\underset{{}_{X_{2}}}{\min}||Y-S(X_{2},\hat{x})|{|}^{2}.$$
  The LM estimation using TSM, Equations (6), (7) were performed iteratively for each image pixel using MATLAB function “lsqcurvefit.” The LM algorithm performs an iterative minimization of the objective function as
  $${\hat{X}}_{i+1}={\hat{X}}_{i}+{\left[J_{i}^{T}J_{i}+\lambda_{i}I\right]}^{-1}[J_{i}^{T}\{Y-S({\hat{X}}_{i})\}],$$
  where $J_{i}=\partial S({\hat{X}}_{i})/\partial {\hat{X}}_{i}$ is the Jacobian matrix of the function S(X) evaluated at the *i*th iteration, I is the identity matrix and *λ_i_* is a scalar. When *λ_i_* is small the minimization is equivalent to a Gauss–Newton algorithm, for large *λ_i_* the minimization tends toward the steepest‐descent algorithm (44). If a step is successful (gives a lower function value), the algorithm sets $\lambda_{i+1}=\lambda_{i}/10$, else it is set as $\lambda_{i+1}=\lambda_{i}\times 10$. In our simulations, the maximum number of iterations was specified as 10 and the other iteration stopping criteria such as the tolerance of step size, size of the gradient and the residuals were left to their default value ( $10^{-6}$).
2. OSM: LM estimation of **X**, solving
  (8) $$\hat{X}=\arg\underset{{}_{X}}{\min}||Y-S(X)|{|}^{2}.$$
  The LM estimation using OSM, Equation (8) were also performed iteratively for each image pixel using MATLAB function “lsqcurvefit.” The maximum number of iterations and stopping criteria were specified same as that for TSM estimation.
3. OSM‐DGN1: DGN estimation of **X** using Equation (8). OSM‐DGN1 estimation was performed iteratively for each image pixel using a DGN algorithm as,
  $${\hat{X}}_{i+1}={\hat{X}}_{i}+s_{i}{\left[J_{i}^{T}J_{i}\right]}^{-1}[J_{i}^{T}\{Y-S({\hat{X}}_{i})\}].$$
  The DGN algorithm utilizes an inexact line search algorithm for estimating *s_i_* 40, 41. It was shown in 41 that the DGN algorithm displayed better convergence and robustness for various choices of initializations than LM for a ill‐posed problem. The maximum number of iterations and stopping criteria for DGN iterations were specified same as the previous methods.
4. OSM‐DGN2: DGN estimation of **X**, solving
  (9) $$\hat{X}=\arg\underset{{}_{X}}{\min}||Y-S(X)|{|}^{2}+F(X).$$

where F(X) is the regularization functional, constructed based on the prior information of the model parameters 45, 46, 47. In this work, we chose $F(X)=\lambda ||L_{X}(X-X_{*})|{|}^{2}$. Here $X_{*}$ is the prior mean, $L_{X}{}^{T}L_{X}=\Gamma_{X}^{-1}$, and $\Gamma_{X}$ = diag( $\sigma_{S_{0}}^{2},\ldots ,\sigma_{V_{6}}^{2}$) is a diagonal (Covariance) matrix of the variances of **X**. We note that the estimate, Equation (9), can be interpreted in the Bayesian inversion framework as the maximum a posteriori estimate from a posterior density model, which is based on the observation model, Equation (4), and a Gaussian prior for the model parameters 20, 48. The prior was constructed based on the reported values of IVIM parameters and the std's were chosen to allow for sufficient variations for detecting different pathological conditions. See Table 1 for the means and variances in our prior. DGN estimation of, Equation (9), was performed iteratively for each pixel as,

$${\hat{X}}_{i+1}={\hat{X}}_{i}+s_{i}{\left[J_{i}^{T}J_{i}\;+\;\lambda \;\Gamma_{X}^{-1}\right]}^{-1}[J_{i}^{T}\{Y-S({\hat{X}}_{i})\}-\lambda \;\Gamma_{X}^{-1}\{{\hat{X}}_{i}-X_{*}\}].$$

**Table 1:** Prior Means and Variances

Parameter	Mean	Standard deviation	References
*S*_0_	Mean ( $S_{0,meas}$)	Std ( $S_{0,meas}$)	
*f*	0.1	0.1	25, 49
$U_{1},\ldots ,U_{6}$	$\sqrt{0.007\;{\text{mm}}^{2}/s}$	$\sqrt{0.005\;{\text{mm}}^{2}/s}$	49
$V_{1},\ldots ,V_{6}$	$\sqrt{0.0007\;{\text{mm}}^{2}/s}$	$\sqrt{0.000025\;{\text{mm}}^{2}/s}$	25

The regularization parameter was manually chosen as $\lambda =10^{-2}\times \text{trace}(J_{i}^{T}J_{i})/\text{trace}(\Gamma_{X}^{-1})$. For systematic approaches of the regularization parameter selection see 50, 51. The maximum number of iterations and stopping criteria for DGN iterations were specified same as the previous methods.

We initialized the parameters such that tensors $D^{*},\;D$ are isotropic 39

(10) $$\begin{array}{l} X_{0}=(S_{0,meas},f_{\text{init}},\sqrt{\text{MD}{\left(D^{*}\right)}_{\text{init}}},\sqrt{\text{MD}{\left(D^{*}\right)}_{\text{init}}},\sqrt{\text{MD}{\left(D^{*}\right)}_{\text{init}}},0,0,0,\\ \sqrt{\text{MD}{\left(D\right)}_{\text{init}}},\sqrt{\text{MD}{\left(D\right)}_{\text{init}}},\sqrt{\text{MD}{\left(D\right)}_{\text{init}}},0,0,0), \end{array}$$

where $X_{0}$ are the initial IVIM‐DTI parameters, $S_{0,meas}$ is the measured noisy *S*_0_ value. The parameters $f_{\text{init}},\;\;\text{MD}{\left(D^{*}\right)}_{\text{init}},\;\text{MD}{\left(D\right)}_{\text{init}}$ are the initial values of *f* and mean diffusivities of $D^{*},\;D$ respectively. To test the estimates with different initial parameter values, we quantify the distance between initial and target values by the ratio

(11) $$X_{\text{init}}=\frac{f_{\text{init}}}{f_{\text{target}}}=\frac{\text{MD}{\left(D^{*}\right)}_{\text{init}}}{\text{MD}{\left(D^{*}\right)}_{\text{target}}}=\frac{\text{MD}{\left(D\right)}_{\text{init}}}{\text{MD}{\left(D\right)}_{\text{target}}}.$$

### Synthetic Data Generation

We have synthetically generated dMRI signals from capillary flow and tissue diffusion for two different configurations. These were later used to evaluate the different estimation methods.

#### Configuration 1

**Figure 1:**
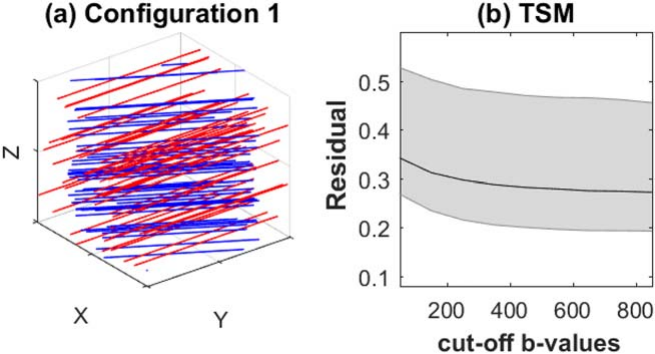
**a**: Configuration 1 of simulated data: the red lines represents vessels oriented along *x*‐axis and the blue lines represents axons oriented 30° to the *x*‐axis on the xy‐plane. **b**: Dependence of residual error, *R*, Equation [15] of TSM estimates with cut‐off *b*‐value, $b_{\text{cut}‐\text{off}}$.

The purpose of the first configuration is to test the estimation methods with known ground‐truth IVIM‐DTI parameters. The *D* ^*^ equivalent of flow has a known analytical expression in a system of identically oriented tubes (mimicking vessels) with Gaussian distributed velocities 22. In the first simulation configuration shown in Figure 1a, we considered such a system with velocities, v∼N (0.86 mm/s, 0.34 mm/s) along *x*‐axis undergoing “plug” flow 52. The vascular dMRI signal, $Y_{\text{vascu}}$ was generated as 22

(12) $$Y_{\text{vascu}}=||\int \exp(-i\gamma m\cdot v)\exp(-bD_{\text{blood}})\;dv\;||$$

where *γ* is the Gyromagnetic ratio, m is a vector along the gradient direction (see^52^), v is a vector of velocity with magnitude *v* and $D_{\text{blood}}$ = 3 × 10^−3^ mm^2^/s is the intrinsic diffusivity of blood. We assumed 20% of the extra‐vascular diffusivity was due to a system of tubes (mimicking axons) with intrinsic diffusivity $D_{\left|\right|}$ = 0.0017 mm^2^/s 4, orientation $\hat{n}={\left(\cos30{}^{\circ},\sin30{}^{\circ},0\right)}^{T}$ and the rest 80% was due to isotropic diffusion $D_{\text{iso}}$=8 × 10^−4^ mm^2^/s due to the extra‐cellular matrix. The extra‐vascular dMRI signal $Y_{\text{diff}}$ was generated as

(13) $$Y_{\text{diff}}=\exp(-b{\hat{g}}^{T}(0.2D_{\left|\right|}{\hat{n}}^{T}\hat{n}+0.8D_{\text{iso}}I)\hat{g}).$$

The measurement vector, **Y** was generated as

(14) $$Y=S_{0}\;(f\;Y_{\text{vascu}}+(1-f)\;Y_{\text{diff}})+e.$$

where *f* was chosen as 0.12, *S*_0_ as 1 and *e* was the added Rician noise 53. A set of 500 realizations of Y with their corresponding *S*_0_ data having SNR ranging from 5 to 50 were computed using 14. Stejskal‐Tanner pulses with duration *δ* = 1.9 ms, separation Δ = 2 ms and 11 *b*‐values (*b* = 0, 50, 150, 250, 350, 450, 550, 650, 750, 850, 1150 s/mm^2^) with 12 *b*_0_ images and 60 non‐co‐linear gradient directions for other shells were used to generate the data.

#### Configuration 2

In the second simulation configuration, we constructed a realistic in silico tissue model in a 1 mm × 1 mm × 2 mm domain as shown in Figure 5. The domain used a Gaussian Markov random field model 54 for the map of *S*_0_ (Fig. 5a), two vascular trees mimicking arterioles and venules and a capillary network connecting them (Fig. 5b), and a distribution of cylinders modeling axons (Fig. 5c).

For generating the capillary network, we used a modified spanning tree algorithm presented in 55. For generating the arteriole and venule trees, we used the VascuSynth software^56^. To compute the vascular dMRI signal, Equation (12), the flow velocity, *v_ij_* connecting node *i* to *j* of a vessel segment was calculated from the volumetric flow rate *Q_ij_* as $v_{ij}=Q_{ij}/\pi r_{ij}^{2}$, where *r_ij_* is the radius of the segment. The *Q_ij_*'s and *r_ij_*'s of the vascular trees were obtained using VascuSynth by specifying a smoothly varying oxygen demand map. The parameters used in VascuSynth are mentioned in Supporting Table S1. For calculating *Q_ij_*'s in the capillary network, we used Poiseuille's equation and pressure conservation 55. The simulated velocities are displayed in Figure 5b.

The simulated axon orientations, as shown in Figure 5c, were drawn using the Watson distribution and a spatially smoothly varying degree of dispersion 4. The intrinsic diffusion coefficients, D ${}_{\left|\right|}$'s in simulated axons were drawn from a Gaussian distribution, D ${}_{\left|\right|}∼N$(0.0017 mm^2^/s, 0.0008 mm^2^/s). The axon lengths were drawn from a Gaussian distribution with mean 10^−4^ m and s.t.d. 10^−5^ m. The voxel size was specified as 0.2 mm × 1 mm × 0.2 mm. The dMRI signal were calculated using Equation (14). The *S*_0_ data had SNR equal to 50. The target $S_{0},\;f$ maps and measures of **D** are shown in Figure 6a. There is no available method (analytical or numerical) to calculate the D^*^ equivalent of the current flow configurations and hence not shown in Figure 6a. Although configuration 2 is more realistic than configuration 1, sensitivity analysis of TSM, OSM, OSM‐DGN1, and OSM‐DGN2 in estimating D^*^ cannot be performed using this configuration, since the target D^*^ is unknown.

### In Vivo Data

A diffusion weighted data set of a healthy volunteer was acquired using a 3T Siemens MAGNETOM Prisma ${}^{\text{Fit}}$ system. An EPI/spin echo (SE) diffusion weighted pulse sequence was used with a 128 × 128 acquisition matrix which resulted in an isotropic voxel size of 2.5 mm. The number of slices was 20. The echo time was set to 75 ms and the pulse repetition time to 2700 ms. The acquisition time was approximately 30 min. The diffusion weighted gradient settings that were used consisted of 11 *b*‐values (*b* = 0, 50, 150, 250, 350, 450, 550, 650, 750, 850, 1150 s/mm^2^) with 12 *b*_0_ images and 60 non‐co‐linear magnetic field gradient directions for the others. The first step in the post‐processing pipe‐line was the denoising of the dMRI data by exploiting its inherent redundancy using random matrix theory^57^. Next, Gibbs ringing correction based on local interpolation in k‐space was applied^58^. The “Topup” 59and “Eddy” 60 tools in FSL were used to correct for susceptibility, eddy current, and subject motion distortions.

## RESULTS

In this section, we present the estimates obtained using TSM, OSM, OSM‐DGN1, and OSM‐DGN2 using the in‐silico phantoms and in‐vivo data. Residual error,

(15) $$R=||Y-S(X)|{|}^{2},$$

along with percentage (relative) errors in estimated *f*, percentage errors in standard tensor measures (MD and FA) of $D^{*},\;D$ and the orientation errors of $D^{*},\;D$. The orientation errors were measured as angles of the major eigenvectors of the estimated tensors to the target tensor orientations 61.

### Evaluation of *f*, $D^{*},\;D$ Estimates and Residual Using Configuration 1

It has been previously observed that IVIM TSM estimates are sensitive to the choice of cut‐off *b*‐values 30. To test the sensitivity of IVIM‐DTI TSM estimation to cutoff *b*‐values the residual error, *R*, was calculated for a series of values. Figure 1b shows that R is nearly constant after *b* = 500 mm^2^/s, which was chosen as the cutoff *b*‐value.

**Figure 2:**
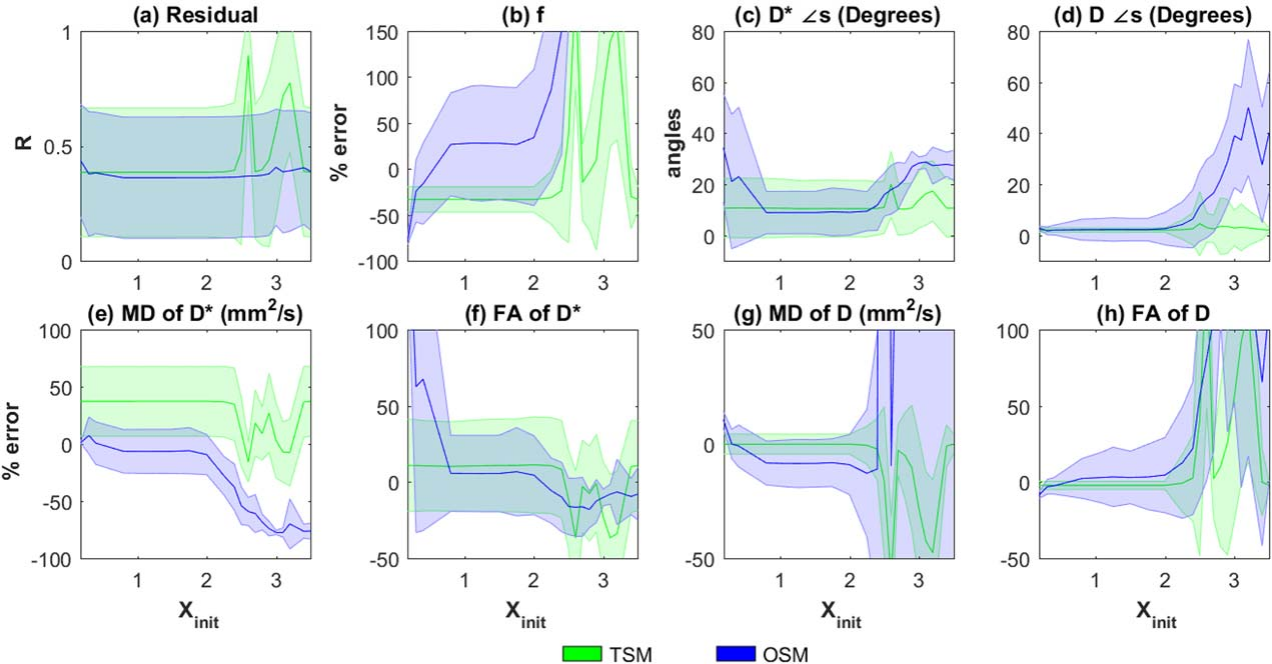
Dependence on initialization on TSM and OSM estimates. Plots of (**a**) R, (**b**) percentage errors in *f*, (**c**) error in angles (in degrees) of estimated D^*^ and (**d**) **D**, percentage errors in (**e**) MD of D^*^, MD(D^*^), (**f**) FA of D^*^ ^,^ FA(D^*^), (**g**) MD of **D**, MD(**D**), (**h**) FA of **D**, FA(**D**) plotted against X_init,_ Equation [11]. In all the plots, the thick lines represent the mean of the estimated values from the 500 noisy samples of **Y** and the shaded region represents the standard deviation. Here X_init_ varies from 0.2 to 3.5.

**Figure 3:**
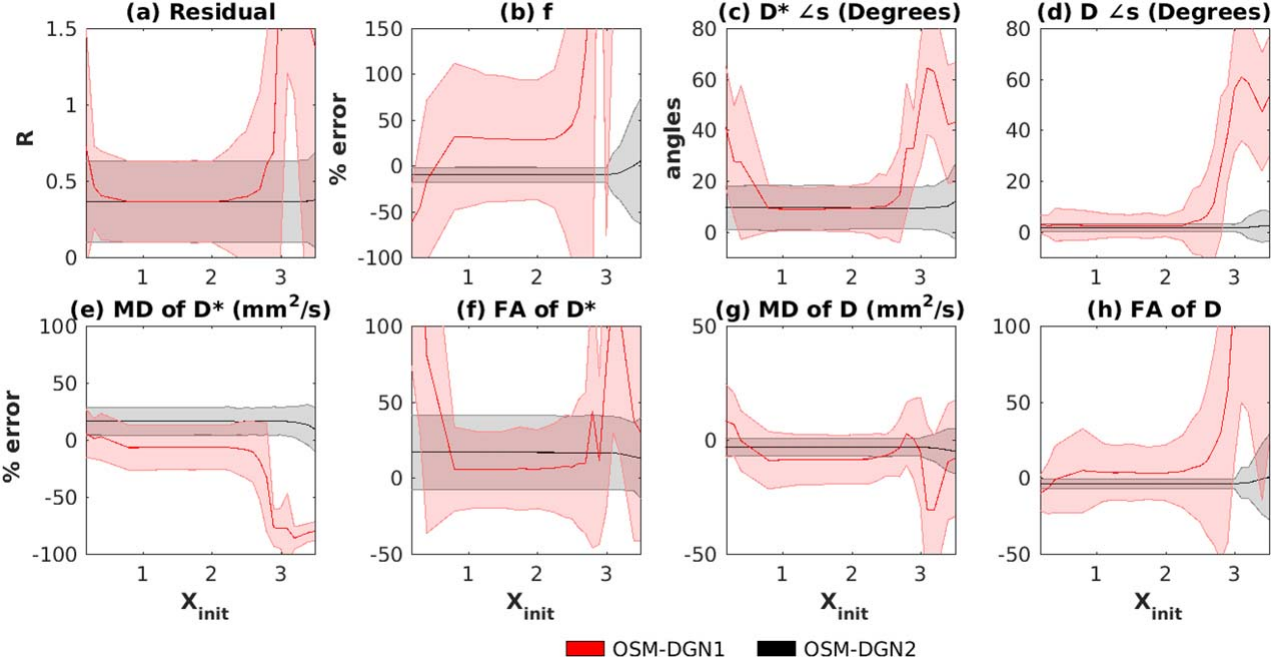
Dependence on initialization on OSM‐DGN1 and OSM‐DGN2 estimates. Plots of (**a**) R, (**b**) percentage errors in *f*, (**c**) error in angles (in degrees) of estimated D^*^, and (**d**) **D**, percentage errors in (**e**) MD of D^*^, MD(D^*^), (**f**) FA of D^*^, FA(D^*^), (**g**) MD of **D**, MD(**D**), (**h**) FA of **D**, FA(**D**) plotted against X_init,_ Equation [11]. In all the plots, the thick lines represent the mean of the estimated values from the 500 noisy samples of **Y** and the shaded region represents the standard deviation. Here X_init_ varies from 0.2 to 3.5.

It is known that OSM estimation is sensitive to the choice of initial‐values 17. Hence, the performances of the estimation methods were evaluated for varying parameter initialization using the generated synthetic data. The results are shown in Figures 2 and 3. Errors in MD(D^*^) appear highest in TSM ( p<10^−20^ for errors of MD $\left(D^{*}\right)$ between TSM and other estimates at X ${}_{\text{init}}=2.5$). OSM showed the most sensitivity to initial values (*p* < 10^−18^ for errors of *f*, and D^*^ measures between OSM and other methods at X ${}_{\text{init}}=3$). DGN methods displayed lower sensitivity to inital values (*p* < 0.02 for *f* between OSM‐DGN1/OSM‐DGN2 and TSM/OSM errors at X ${}_{\text{init}}=3$). OSM‐DGN2 displayed lower errors compared to OSM‐DGN1 ( $p<10^{-15}$ for errors of *f* and $D^{*},D$ measures between OSM‐DGN1 and OSM‐DGN2 at X ${}_{\text{init}}=2.5$).

**Figure 4:**
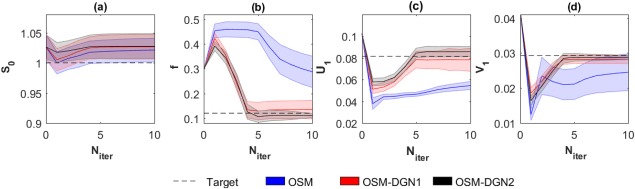
Convergence and uncertainties of OSM, OSM‐DGN1 and OSM‐DGN2 estimates of (**a**) *S*_0_, (**b**) *f*, (**c**) *U*_1_, and (**d**) *V*_1_ with iteration number, N ${}_{\text{inter}}$. The mean and standard deviations of the estimates obtained with 500 noisy samples of data **Y** are shown. The initial value, X_init_ was 2.5.

Figure 4 displays the convergence of OSM, OSM‐DGN1, and OSM‐DGN2 estimates with iteration number, $N_{\text{iter}}$. It can be seen that OSM‐DGN1 and OSM‐DGN2 converge faster than OSM.

### Evaluation of *f*, D Estimates and Residual Using Configuration 2

**Figure 5:**
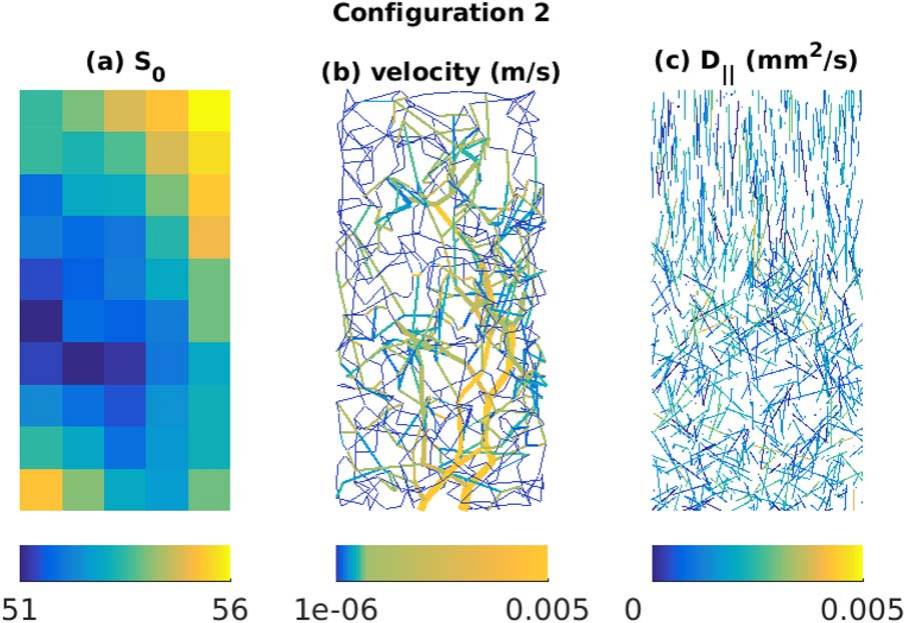
Configuration 2 of simulated data. The images are displayed along *x‐z* plane of the three‐dimensional (1 mm × 1 mm × 2 mm) domain. **a**: Simulated *S*_0_ map using a Gaussian Markov random field model. **b**: Synthetic vascular network of arterioles, venules and capillaries using the VascuSynth software and modified spanning tree algorithm (MSTM). **c**: Distribution of cylinders representing axons. The axon orientations were drawn using the Watson distribution.

**Figure 6:**
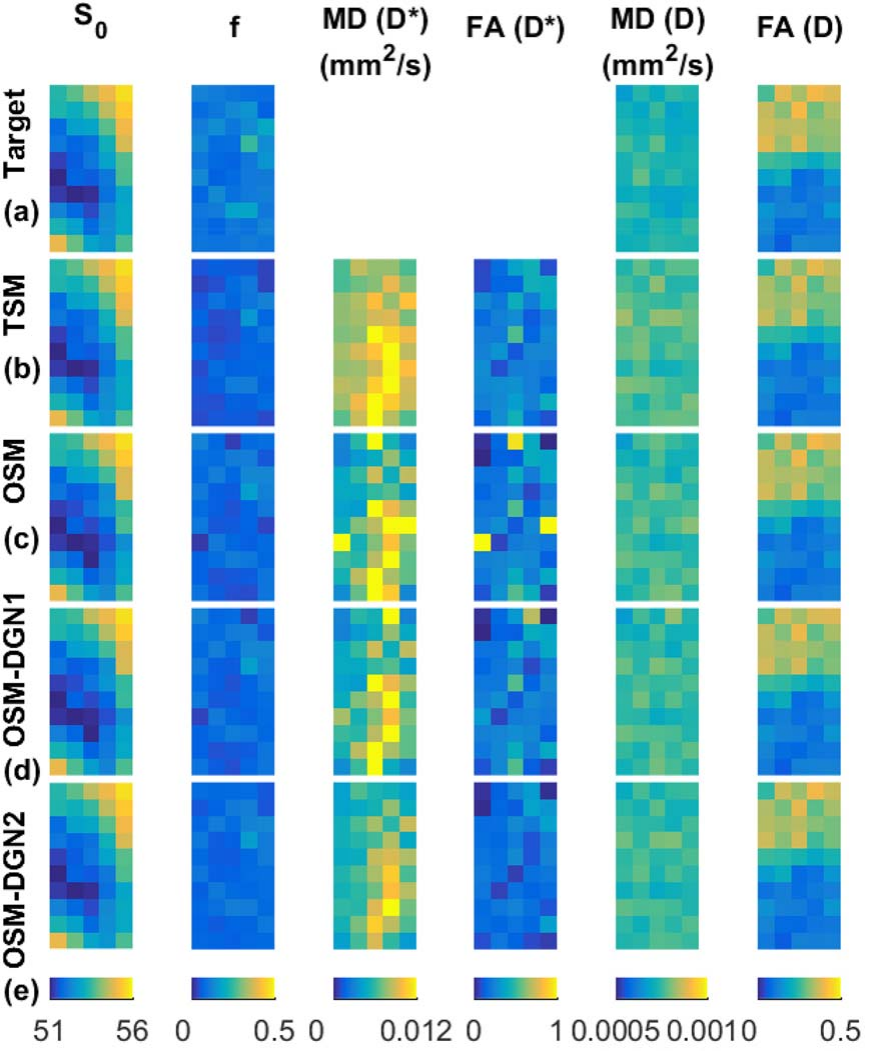
Reconstructions of IVIM‐DTI parameters using Configuration 2, Figure 5. The *S*_0_, *f*, MD(D^*^), FA(D^*^), MD(D^*^), FA(D^*^) maps of the (**a**) Target, (**b**) TSM, (**c**) OSM, (**d**) OSM‐DGN1, (**e**) OSM‐DGN2 estimates are shown. Note that MD(D^*^), FA(D^*^) of the target are unknown.

**Figure 7:**
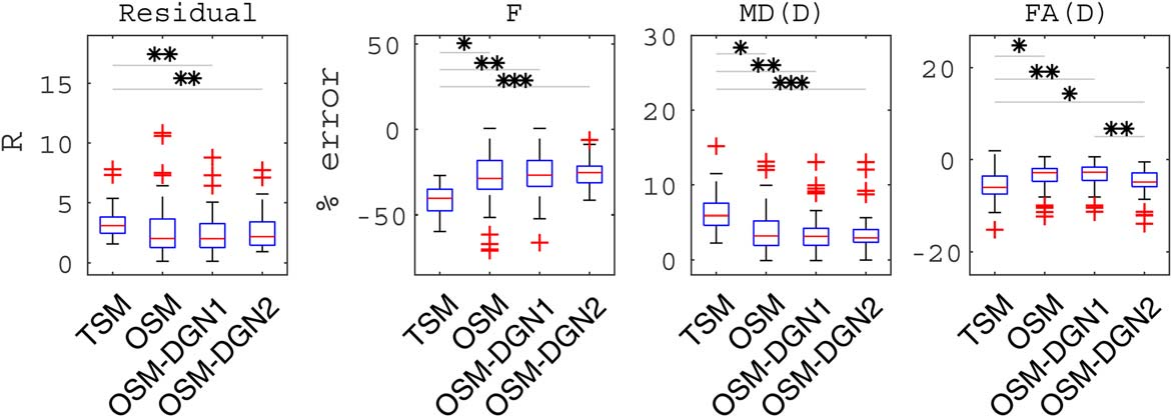
Reconstructions of IVIM‐DTI parameters using Configuration 2, Figure 5. The residual R, percentage errors in estimates of *f*, MD(**D**), FA(**D**) for the four methods are shown along with the *p*‐values (obtained by applying the student's t‐test between the different methods) represented with star symbols (*). Here we represent the *p*‐values as: * for *p* < 10^−2^, ** for *p* < 10^−5^ and ∗∗∗ for *p* < 10^−10^.

The target IVIM‐DTI parameter maps of simulated domain, displayed in Figure 5, are shown in Figure 6a. The estimated parameter maps using the different methods are shown in Figure 6b–d. The initial value of **X** was chosen as $f_{\text{init}}$ = 0.02, MD ${\left(D^{*}\right)}_{\text{init}}$ = 0.005 mm^2^/s, MD ${\left(D\right)}_{\text{init}}$ = 0.0005 mm^2^/s, which corresponded roughly to the mean target values. Given that the initial values were close to target values, only a few pixels of OSM estimated parameter maps showed deviations from the target parameter maps. Residual *R* and percentage errors in *f*, MD(**D**), and FA(**D**) are shown in Figure 7. OSM‐DGN2 presents the smallest error dispersion for all the parameters.

### Evaluation of Residual Using In Vivo Data

**Figure 8:**
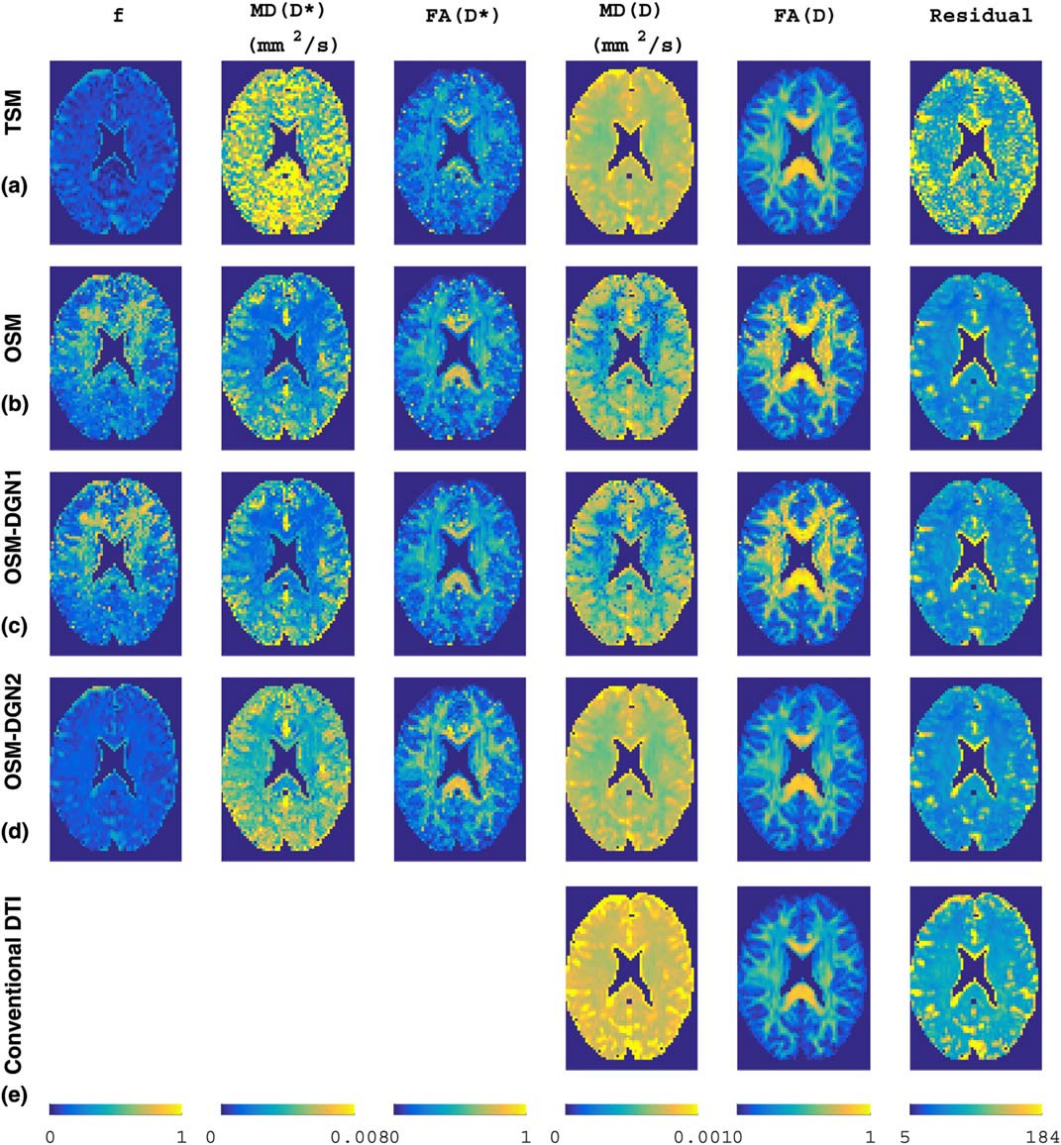
Estimates with in vivo brain dMRI data. **a**: TSM estimates, **b**: OSM estimates, **c**: OSM‐DGN1 estimates, **d**: OSM‐DGN2 estimates, **e**: Conventional DTI estimates. From left: Vascular volume fraction *f*, MD and FA of pseudo diffusion tensor D^*^ ^,^ MD and FA of diffusion tensor **D**, and map of residual error *R*.

**Figure 9:**
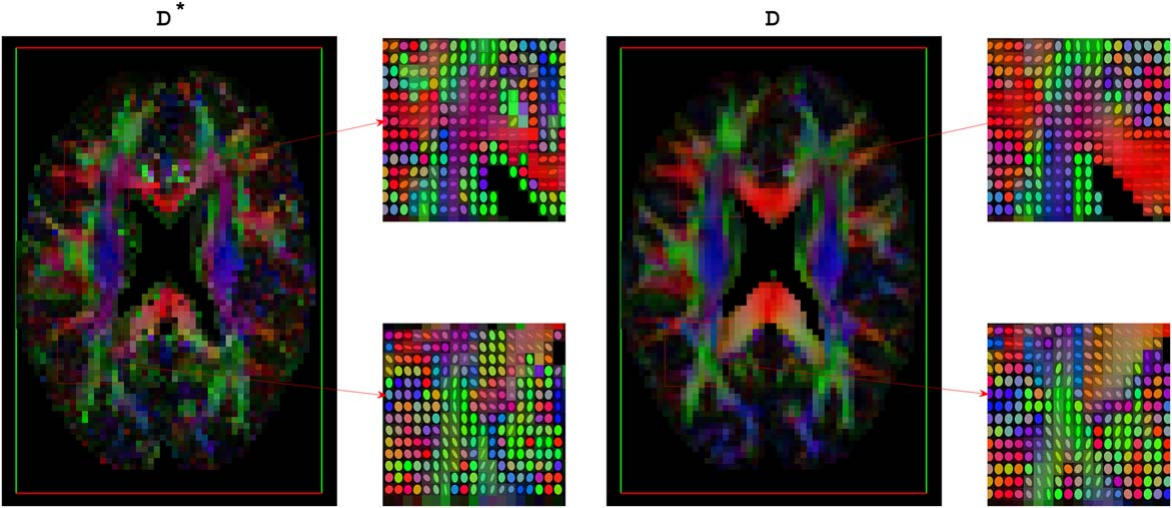
Maps of RGB‐encoded alignments of estimated D^*^ and **D** using OSM‐DGN2, plotted with ExploreDTI.

The TSM, OSM, OSM‐DGN1, and OSM‐DGN2 methods were applied to the in vivo brain dMRI data (see Fig. 8 and Supporting Fig. S2). **X** was initialized as $f_{\text{init}}$ = 0.2, MD ${\left(D^{*}\right)}_{\text{init}}$ = 0.003 mm^2^/s, MD ${\left(D\right)}_{\text{init}}$ = 0.0006 mm^2^/s by fitting the average dMRI signal over all pixels with the IVIM model, Equation [1]. TSM, OSM, OSM‐DGN1, and OSM‐DGN2 estimates of *f*, and measures of $D^{*},\;D$ are shown in Figure 8a–d. The data residuals in the image and their histograms are also shown. We added a row of conventional DTI measures estimated using software TORTOISE version 2.5.1 62 in Figure 8d. The estimated D^*^ and **D** using OSM‐DGN2 were used to generate tensor maps with software ExploreDTI version 4.8.6 63, shown in Figure 9.

## DISCUSSION

The aim of this study was to explore methods to estimate IVIM‐DTI parameters allowing simultaneous pseudo‐diffusion and DTI, and subsequently, finding a robust estimation method. Previously known IVIM approaches were applied to IVIM‐DTI estimation. The influence of initial parameters and convergence of the methods was also studied using simulated data. In our simulations (and in vivo experiment), we employed a wide range of *b*‐values (0 →1150 s/mm^2^) and gradient directions since IVIM‐DTI estimation requires a larger data set (due to larger number of model parameters) compared to standard IVIM/DTI. The first simulated dataset had low SNR (SNR 5 →50 for *S*_0_ data) as in typical clinical scanners 64, and known target IVIM‐DTI parameters. The second dataset had high SNR (SNR = 50 for *S*_0_ data, comparable to high T MRI scanners 64) with more realistic geometry and partially known target IVIM‐DTI parameters. It is known that measurement noise can cause errors in the tensor estimates leading to nonpositive‐definite tensors, particularly in regions of high anisotropy such as the corpus callosum and the corticospinal tract 43. Hence, we enforced positive‐definite constraints on D^*^ and **D**.

In Figure 2 we show the errors in conventional TSM estimates 39 for configuration 1, with different choices of initial values. It can be seen that MD(**D**) appears slightly better estimated using TSM compared to OSM and OSM‐DGN1. This is because OSM estimation is ill‐conditioned and is more affected by measurement noise compared to **D** estimation in TSM. However, the errors in MD(D^*^ ^)^ appear significantly higher in TSM (*p* <10^−20^ at X_init_ = 2.5) indicating D^*^ is most negatively affected in TSM. The TSM estimates with a realistic phantom, Figures 6b and 7 again display higher estimation errors (statistically significant in all measures) compared to other methods. In the context of disease detection and treatment, changes in IVIM parameters (*f*, *D* ^*^ equivalent to MD(D^*^), *D* equivalent to MD(**D**)) have been reported for several diseases in 26, 65, 66, and, recently in breast cancer treatment 67. Figures 2, 6, and 7 shows how utilizing TSM would lead to high estimation errors and could make estimation of such parameter changes unfeasible in clinical settings where low SNR data is available.

In this work, we extended the conventional LM estimation of IVIM parameters to one‐step IVIM‐DTI parameter estimation. It can be seen in Figures 2 and 3, that OSM show relatively lower errors than TSM for D^*^ measures around X ${}_{\text{init}}=1$, however it deteriorates fast with higer/lower initializations and low SNR data compared to other methods (*p* <10^−18^ at X_init_ = 3). This sensitivity to initialization and noise is due to the ill‐conditioned nature of the OSM problem 17.

A new OSM based on weighted NLLS estimation of IVIM‐DTI parameters was also proposed and tested. The method was based on the DGN method with a line search 40, 41. Figure 3a–h demonstrates that the proposed DGN methods, particularly OSM‐DGN2, displays lower sensitivity to initial values compared to LM based TSM and OSM methods. In Figure 3 OSM‐DGN2 show more accurate estimates (with low SNR and incorrect initialization) compared to OSM‐DGN1 (*p* < 10^−15^ at X_init_ = 2.5). This is because the regularizer F(X) stabilises the *ill*‐conditioned problem, Equation (8) by introducing prior information about the model parameters, resulting in more stable and accurate OSM estimates^48^. Figure 4 shows that DGN converges with fewer iterations and estimates parameters more accurately than LM. This is expected, since in the neighborhood of the solution, the LM scalar *λ_i_* is progressively smaller in each iteration and it is equivalent to a regular GN iteration 44. DGN converges with fewer iterations since every update explicitly optimizes the step‐length, instead of taking a constant as in regular GN iterations. The dMRI signal is affected by additive Gaussian noise in the real and imaginary parts, which results in a signal amplitude with Rician noise. Rician noise is characterized by a skew distribution with non‐zero positive mean. Hence, the assumption of zero‐mean Gaussian distribution in the NLLS estimation leads to a data misfit as observed in Figure 4.

All four estimation methods were applied to real dMRI data. Figure 8 a shows that MD(**D**), FA(**D**) reflect known microstructural features for such brain regions from previously published DTI studies 68. However, *f* and measures of D^*^ and **D** in Figure 8b–d display different characteristics compared to TSM maps in Figure 8a. The higher residual errors in TSM estimates compared to other estimates in Figure 8 along with poorly estimated *f* and D^*^ measures in Figures 2 and 7 suggest that the physical assumption of pure diffusion contribution at high *b*‐values could lead to slightly higher than actual estimated values of **D**, and subsequently erroneous *f* and D^*^ estimates. Such assumptions are not present in OSM. Nevertheless, the OSM methods displays high sensitivity to noise and initial values 24, 49. The very low MD(D^*^), MD(**D**) and high *f* seen in frontal white matter in Figure 8b,c, seen also in simulated low SNR data in Figures 2 and 3, is possibly due to wrong fitting due to ill‐conditioning and low SNR in these regions 64. The low pseudo‐diffusion contribution ( f≈ 0.1) is partially masked by the noise and in the absence of prior conditioning, the data fits the bi‐tensor model to the diffusion signal alone, leading to lower than usual MD(D^*^) and MD(**D**) values. As seen in Figure 2, OSM‐DGN2 is more robust to noisy data due to prior conditioning of the problem and the *f*, MD(D^*^) and **D** maps in Figure 8d match previously reported brain IVIM maps 25, 49. In most configurations, dMRI signal rapidly changes with flow (or perfusion) variations 22, 37, 52. This is possibly why the estimated measures of D^*^ from simulations (Fig. 5) and in vivo data (Fig. 8) are grainy/nonsmooth compared to **D**. The high residuals near the CSF boundaries and in deep gray matter are due to partial volume effects due to CSF and perivascular spaces which shows relatively higher signal intensities 69. The computational times, $t_{\text{CPU}}$s of these estimates were $t_{\text{CPU}}=207$s for TSM, $t_{\text{CPU}}=1259$s for OSM, $t_{\text{CPU}}=1531$s for OSM‐DGN1 and $t_{\text{CPU}}=1748$s for OSM‐DGN2 for the entire slice. The additional computational cost incurred by the line search in DGN represents around a 40% increase. This could be acceptable compared with the advantage in the accuracy of DGN over LM. These results confirm the previous comparison study of LM and DGN on a *ill‐posed* problem in 41 and on the use of prior densities in IVIM estimation 31, 32. Estimates from two slices near “circle of Willis,” demonstrating marked differences between FA(D^*^) and FA(**D**) are also shown in Supporting Fig. S2.

Figure 9 displays the alignment of D^*^ and **D**. One of the striking features of this image is the similarity of the alignments of D^*^ and **D**. Pseudo‐diffusion is directly related to perfusion 14, hence, MD(D^*^) and alignment resembles mean perfusivity and perfusion tensor alignments reported in recent arterial spin labeling based PTI brain study 70. The alignment of the D^*^ in the white matter is also in agreement to previously reported white matter vascular tract studies 71 and white matter IVIM studies 72. The simulation results in Figure 2c,d, which demonstrate the capability of OSM‐DGN2 in efficiently estimating D^*^ and **D** orientations, along with the aforementioned studies 70, 71, 72, suggest that Figure 9 is indeed a map of capillary microarchitecture orientations. This is the first time such a map of the human brain has been shown using dMRI.

In this work, we only compared NLLS approaches. Markov chain Monte Carlo methods were reported to demonstrate higher precision and accuracy compared to NLLS in IVIM studies in 17, 28. Although TSM and OSM estimation could be extended to Markov chain Monte Carlo ‐based methods, in high‐dimensional problems such as IVIM‐DTI, Markov chain Monte Carlo methods are computationally intensive and unsuitable for fast online estimation in clinical settings. Also, to our knowledge, most popular DTI softwares are based on NLLS estimation 73. Hence, existing NLLS DTI codes could be easily modified for IVIM‐DTI using the proposed OSM‐DGN2 approach.

One‐step joint estimation of pseudo‐diffusion and diffusion tensors using IVIM‐DTI has not been previously carried out. In this work, we introduce the IVIM‐DTI OSM and test it with simulations and one in vivo dMRI study. We also suggest a modification to the OSM using DGN and a Gaussian prior for the model parameters. We also give evidence, for the first time, that brain pseudo‐diffusion aligns to brain diffusion tensors. Pseudo‐diffusion tensor alignments has never been reported using dMRI before. The MATLAB DGN codes used in this article are available for download at 74.

The results indicate that pseudo‐diffusion tensor can be efficiently estimated along with the diffusion tensor using OSM‐DGN2. Popularly used single exponential (DTI) models, which capture only microstructural information, fail to capture information regarding blood microcirculation. Estimating pseudo‐diffusion helps capture blood microcirculation 14, which is vital for the early diagnosis of several disorders such as vascular cognitive impairment 12. Hence, simultaneous estimation of both D^*^ and **D** will provide more information about disorders such as dementia than **D** alone. One drawback of IVIM‐DTI is the requirement of high dimensional data (more *b*‐values and gradient directions) as compared to DTI/IVIM. Future work will address the evaluation of the proposed technique to early differential diagnosis of dementia. In particular, we will seek to associate different stages of degeneration in the vascular and axonal microarchitecture with the relative values and orientation of the diffusion and pseudo‐diffusion tensors.

## CONCLUSIONS

The OSM‐DGN2 show better tolerance to initial values compared to conventionally used LM algorithm and is capable of estimating pseudo‐diffusion and diffusion tensors with less than twenty percent errors in the tensor measures, and less than ten degree errors in the tensor orientations in low SNR dMRI data. Based on our work, we propose a one‐step method based on DGN and Gaussian prior for the model parameters (OSM‐DGN2) for simultaneous diffusion and pseudo‐diffusion magnetic resonance imaging using a IVIM‐DTI model and dMRI techniques.

## Supporting information


**Table S1**. Configuration 2 simulation details.
**Fig. S1**. Histogram of residual error R from (a) TSM estimates, (b) OSM estimates, (c) OSM‐DGN1 estimates, (d) OSM‐DGN2 estimates, and (e) conventional DTI estimates.
**Fig. S2**. Estimated IVIM‐DTI parameters and measures using in vivo dMRI data from two slices (a) and (b) near the “circle of Willis” using IVIM‐DTI2. A marked difference between FA(**D** ${}^{*}$) and FA(**D**) is noticed, possibly due to the presence of a substantial vascular component near the circle of Willis.
**Fig. S3**. Map of voxels showing higher DTI residuals, Res ${}_{\text{DTI}}$ (computed using software TORTOISE version 2.5.1) compared to IVIM‐DTI residuals, Res ${}_{\text{IVIM}-\text{DTI}}$ (computed using OSM‐DGN2). From left to right: map of voxels with (a) Res ${}_{\text{DTI}}>$Res ${}_{\text{IVIM}-\text{DTI}}$, (b) Res ${}_{\text{DTI}}>$1.2 × Res ${}_{\text{IVIM}-\text{DTI}}$, and (c) Res ${}_{\text{DTI}}>$ 1.5 × Res ${}_{\text{IVIM}-\text{DTI}}$. The pixels with highest deviation are seen in the gray matter, possibly due to the presence of more capillaries and higher contribution to pseudo‐diffusion.
