# Supplementary material for: Simultaneous magnetic resonance diffusion and pseudo‐diffusion tensor imaging
Source: Magn Reson Med. 2017 Jul 16;79(4):2367–78. doi: 10.1002/mrm.26840 (PMC5836966; doi:10.1002/mrm.26840)

Supporting Table S1: Configuration 2 simulation details.

VascuSynth parameters	Values	References
Flow rates in arterioles and venules	0.3 m^3/Kg·hr	Obrist *et al.* Circ Res 20(1), 124-135.
Arteriole inlet and outlet pressures	20mmHg & 7.5 mmHg	Su *et al.* Microcirculation 19(2), 175-187.
Venule inlet and outlet pressures	10mmHg & 6 mmHg	
Number of arteriole and venule nodes	50	
**MSTM parameters**	**Values**	
Inlet and outlet pressures	7.5mmHg & 6 mmHg	
Sampled random points	500	
Simulated number of edges	691	
Capillary radii, r_{ij}	$\mathcal{N}(3.12\mu m, 0.6\mu m)$	Su *et al.* Microcirculation 19(2), 175-187.

Supporting Figure S1: Histogram of residual error R from (a) TSM estimates, (b) OSM estimates, (c) OSM-DGN1 estimates, (d) OSM-DGN2 estimates, and (e) conventional DTI estimates.

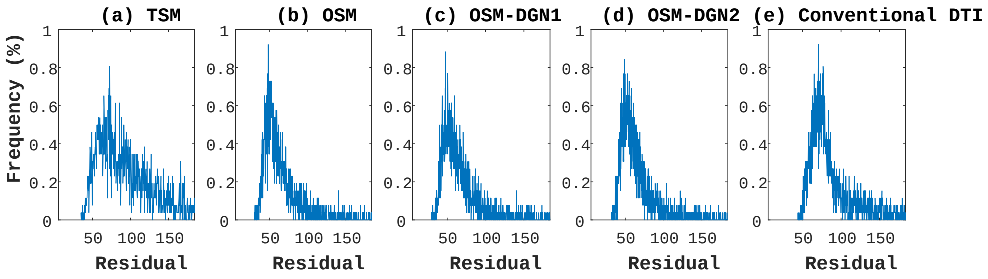

Supporting Figure S2: Estimated IVIM-DTI parameters and measures using *in vivo* dMRI data from two slices (a) and (b) near the "circle of Willis" using IVIM-DTI2. A marked difference between FA($\mathbf{D}^*$) and FA($\mathbf{D}$) is noticed, possibly due to the presence of a substantial vascular component near the circle of Willis.

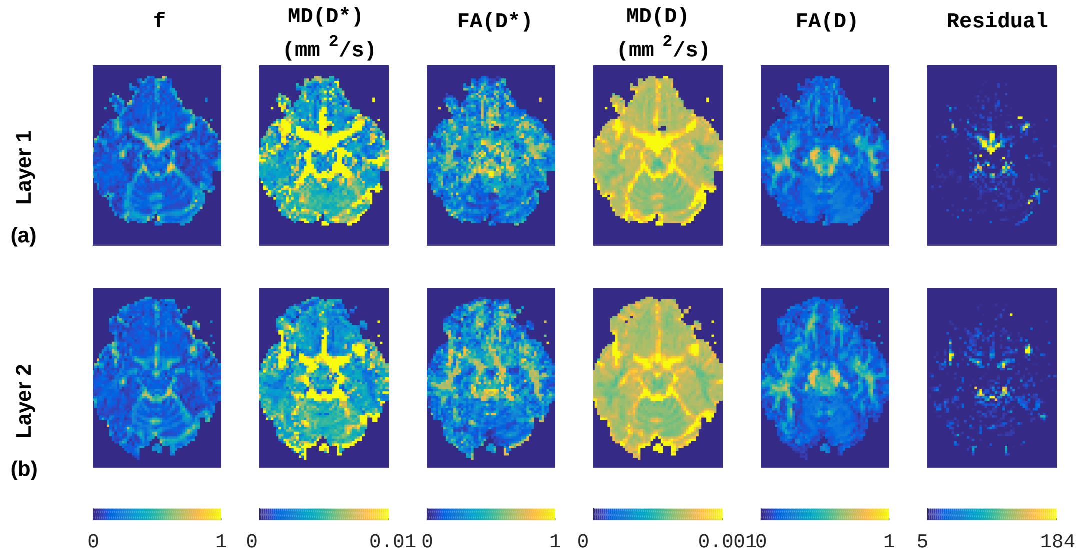

Supporting Figure S3: Map of voxels showing higher DTI residuals, Res_{DTI} (computed using software TORTOISE version 2.5.1) compared to IVIM-DTI residuals, $\text{Res}_{\text{IVIM-DTI}}$ (computed using OSM-DGN2). From left to right: map of voxels with (a) $\text{Res}_{\text{DTI}} > \text{Res}_{\text{IVIM-DTI}}$, (b) $\text{Res}_{\text{DTI}} > 1.2 \times \text{Res}_{\text{IVIM-DTI}}$, and (c) $\text{Res}_{\text{DTI}} > 1.5 \times \text{Res}_{\text{IVIM-DTI}}$. The pixels with highest deviation are seen in the gray matter, possibly due to the presence of more capillaries and higher contribution to pseudo-diffusion.

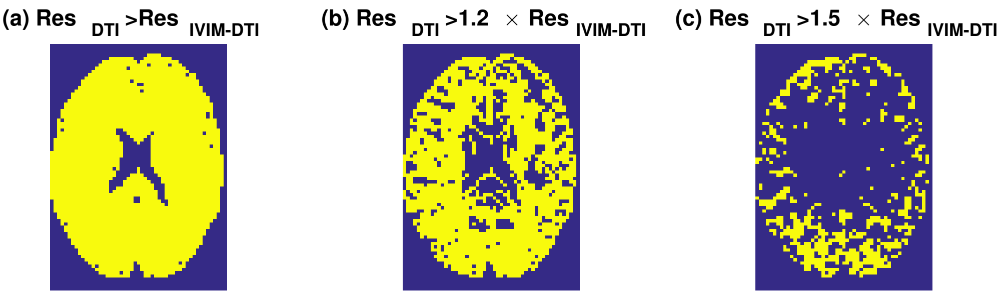

Supplement: Supplementary file 1 — Table S1. Configuration 2 simulation details. Fig. S1. Histogram of residual error R from (a) TSM estimates, (b) OSM estimates, (c) OSM‐DGN1 estimates, (d) OSM‐DGN2 estimates, and (e) conventional DTI estimates. Fig. S2. Estimated IVIM‐DTI parameters and measures using in vivo dMRI data from two slices (a) and (b) near the “circle of Willis” using IVIM‐DTI2. A marked difference between FA(D ∗) and FA(D) is noticed, possibly due to the presence of a substantial vascular component near the circle of Willis. Fig. S3. Map of voxels showing higher DTI residuals, Res DTI (computed using software TORTOISE version 2.5.1) compared to IVIM‐DTI residuals, Res IVIM−DTI (computed using OSM‐DGN2). From left to right: map of voxels with (a) Res DTI>Res IVIM−DTI, (b) Res DTI>1.2 × Res IVIM−DTI, and (c) Res DTI> 1.5 × Res IVIM−DTI. The pixels with highest deviation are seen in the gray matter, possibly due to the presence of more capillaries and higher contribution to pseudo‐diffusion. [file MRM-79-2367-s001.pdf]
